# Loss-of-Function *PTPRD* Mutations Lead to Increased STAT3 Activation and Sensitivity to STAT3 Inhibition in Head and Neck Cancer

**DOI:** 10.1371/journal.pone.0135750

**Published:** 2015-08-12

**Authors:** Noah D. Peyser, Yu Du, Hua Li, Vivian Lui, Xiao Xiao, Timothy A. Chan, Jennifer R. Grandis

**Affiliations:** 1 Department of Otolaryngology, University of Pittsburgh School of Medicine, Pittsburgh, PA, United States of America, 15213; 2 Department of Pharmacology and Chemical Biology, University of Pittsburgh School of Medicine, Pittsburgh, PA, United States of America, 15213; 3 School of Medicine, Tsinghua University, Beijing, China, 100084; 4 Pharmacogenomics and Precision Therapeutics Laboratory, Department of Pharmacology and Pharmacy, The University of Hong Kong, Hong Kong SAR, China; 5 Department of Biochemistry, The University of Hong Kong, Hong Kong SAR, China; 6 Human Oncology and Pathogenesis Program and Department of Radiation Oncology, Memorial Sloan-Kettering Cancer Center, 1275 York Avenue, New York, NY, United States of America, 10065; 7 Department of Otolaryngology, University of California San Francisco, San Francisco, CA, United States of America, 94143; The Chinese University of Hong Kong, HONG KONG

## Abstract

**Background:**

Protein tyrosine phosphatase receptor type D (PTPRD) is a putative tumor suppressor in several cancers including head and neck squamous cell carcinoma (HNSCC). STAT3 is a frequently hyperactivated oncogene in HNSCC. As STAT3 is a direct substrate of PTPRD, we sought to determine the genetic or epigenetic alterations of *PTPRD* that contribute to overactive STAT3 in HNSCC.

**Methods:**

We analyzed data from The Cancer Genome Atlas (TCGA) and our previous whole-exome sequencing study and summarized the mutation, methylation, and copy number status of *PTPRD* in HNSCC and other cancers. *In vitro* studies involved standard transfection and MTT protocols, as well as methylation-specific PCR.

**Results:**

Our findings indicate that *PTPRD* mutation, rather than methylation or copy number alteration, is the primary mechanism by which PTPRD function is lost in HNSCC. We demonstrate that overexpression of wild-type PTPRD in HNSCC cells significantly inhibits growth and STAT3 activation while PTPRD mutants do not, suggesting that mutation may lead to loss of function and subsequent hyper-phosphorylation of PTPRD substrates, especially STAT3. Importantly, we determined that HNSCC cells harboring an endogenous *PTPRD* mutation are more sensitive to STAT3 blockade than *PTPRD* wild-type cells. We additionally found that *PTPRD* mRNA expression does not correlate with pSTAT3 expression, suggesting that alterations that manifest through altered mRNA expression, including hypermethylation and gene copy number alterations, do not significantly contribute to STAT3 overactivation in HNSCC.

**Conclusion:**

*PTPRD* mutation, but not methylation or copy number loss, may serve as a predictive biomarker of sensitivity to STAT3 inhibitors in HNSCC.

## Introduction

Protein tyrosine phosphatase receptor type D (PTPRD) is a member of the receptor-type protein tyrosine phosphatase (PTPR) family. PTPRs are membrane-integral enzymes that catalyze the removal of phosphate groups from specific proteins, thereby impacting cell signaling. Dysregulation of PTPR signaling by genetic and/or epigenetic mechanisms may therefore ultimately lead to cancerous phenotypes. [1] Several PTPR family members, including PTPRD, have been reported to function as tumor suppressors where loss of function alterations may drive tumor growth. [2,3] Genetic events including mutation, gene deletion, or epigenetic silencing may lead to decreased phosphatase activity of PTPRs and enhanced oncogenic signaling. [1]

We recently reported the cumulative mutation profile of the *PTPR* gene family in cancer with a focus on *PTPRT* mutation leading to STAT3 activation in head and neck squamous cell carcinoma (HNSCC). [4] Our analysis revealed that 15 solid tumor types harbored mutations of at least one *PTPR* gene. *PTPRD* is one of the most commonly mutated *PTPR* family members in HNSCC, and PTPRD has been reported to function as a tumor suppressor in this malignancy. [5] *PTPRD* is also mutated, deleted, or hyper-methylated in glioblastoma (GBM), while the gene is unmethylated and expressed in normal brain tissue. [6] Furthermore, *PTPRD* mutations were found to be associated with increased expression of phosphorylated STAT3, a direct PTPRD substrate, in GBM. In addition to GBM, 13% and 25% of HNSCC tumors analyzed in the above study harbored *PTPRD* mutation or promoter methylation, respectively. Homozygous deletion of *PTPRD* has additionally been reported in laryngeal cancer, suggesting that genetic aberrations affecting PTPRD function may be a common event across many cancers. [5]

These cumulative findings led us to hypothesize that genetic and/or epigenetic alteration of *PTPRD* may contribute to enhanced signaling and growth in HNSCC where key components of the pathway may serve as plausible therapeutic targets. Here, we summarize the genetic and epigenetic profile of *PTPRD* in HNSCC from The Cancer Genome Atlas (TCGA) and our prior HNSCC mutational landscape study. [7] We then tested the consequences of *PTPRD* alterations found in human HNSCC tumors in relevant preclinical models to assess STAT3 activity and sensitivity to STAT3 inhibition.

## Materials and Methods

### Cell culture, drug treatment, and transfection

All HNSCC cell lines were genotypically verified as previously described. [4] Cal27 cells were obtained from ATCC (Manassas, VA). PE/CA-PJ34clone12 and PE/CA-PJ49 cells were obtained from Sigma-Aldrich (St. Louis, MO). 686LN cells were obtained from Georgia Chen at MD Anderson Cancer Center (Houston, TX). Cal27 cells were cultured in Dulbecco's modified Eagle's medium (DMEM) (Mediatech, Inc., Manassas, VA) supplemented with 10% fetal bovine serum (FBS) (Gemini Bio-Products, West Sacramento, CA). 686LN cells were cultured in DMEM/F12 (Life Technologies, Grand Island, NY) supplemented with 10% FBS. PE/CA-PJ34clone12 and PE/CA-PJ49 were cultured in Iscove's Modification of DMEM (Mediatech Inc., Manassas, VA) supplemented with 10% FBS and 2 mM L-glutamine (Life Technologies, Grand Island, NY). All cells were maintained in an incubator at 37°C and 5% CO_2_. JSI-124 (Calbiochem, Billerica, MA) was dissolved in DMSO. Transfection was performed with Lipofectamine 2000 (Life Technologies, Grand Island, NY) or FuGENE HD (Promega Corporation, Madison, WI) according to the manufacturer’s instructions with 4 μg DNA diluted in Opti-MEM (Life Technologies, Grand Island, NY).

### Site-directed mutagenesis

*PTPRD* mutations (K1502M, S384R, T1100M and L1147F) were generated from the wild-type plasmid using the Phusion site-directed mutagenesis kit (Thermo Fisher Scientific, Waltham, MA) following the manufacturer’s protocol and confirmed by Sanger sequencing. Plasmid amplifications were performed using the QIAprep Spin Miniprep Kit (Qiagen, Hilden, Germany) or the Hurricane Maxi Prep Kit (GerardBIOTECH, Oxford, OH) according to the manufacturer’s instructions.

### Immunoblotting

Primary antibodies for pSTAT3 (Y705) and STAT3 were obtained from Cell Signaling Technology (Danvers, MA). β-tubulin primary antibody was purchased from Abcam (Cambridge, MA). Secondary antibodies were purchased from BioRad (Hercules, CA). Blots were quantitated by densitometry using ImageJ software.

### MTT assay

MTT assays were performed by incubating cells in 5 mg/ml 3-[4,5-dimethylthiazol-2-yl]-2,5 diphenyl tetrazolium bromide in PBS at for 30 min at 37°C and 5% CO_2_. After incubation, the solution was aspirated and replaced with DMSO. Absorbance of this solution was measured at 570 nm on a spectrophotometer.

### Methylation-specific PCR

Tumor and normal tissue were obtained under the auspices of an Institutional Review Board-approved protocol at the University of Pittsburgh. DNA was isolated from formalin-fixed paraffin embedded tissue using the QIAamp DNA FFPE Tissue Kit, and cell line DNA was isolating using the QIAamp DNA Mini Kit. Bisulfite conversion was performed using the EpiTect Bisulfite Kit and methylation-specific polymerase chain reaction (MSP) was conducted using EpiTect MSP Kit. All kits were used according to the manufacturer’s instructions (Qiagen, Hilden, Germany). MSP primers were designed using MethPrimer software. [8] For methylation, the forward and reverse primer sequences were GTTTTATTTTGGATTTTTGGAATC and TACCTAACGCGACCTAAACG, respectively. For unmethylation, the forward and reverse primer sequences were TATTTTGGATTTTTGGAATTGT and AACTACCTAACACAACCTAAACAAA, respectively. Primers were specific for the intended methylation status as determined using the EpiTect Control DNA Set (Qiagen, Hilden, Germany).

### Data download and analysis

Mutation, copy number alteration, and RNA-Seq data were aggregated from the cBio Portal [9,10] and published reports. [6,7,9] DNA methylation data were obtained through the TCGA data matrix (https://tcga-data.nci.nih.gov/tcga/dataAccessMatrix.htm). DNA and protein sequences and domain annotations were obtained from the National Center for Biotechnology Information (http://www.ncbi.nlm.nih.gov). Reverse phase protein array data were obtained from The Cancer Proteome Atlas (http://app1.bioinformatics.mdanderson.org/tcpa/_design/basic/index.html). Statistical tests were performed using GraphPad Prism 5 software (GraphPad, La Jolla, CA).

### Trypan Blue Exclusion

PE/CA-PJ34clone12 cells were plated on 6-well plates at 100,000 cells per well. After 24 hours, cells were transfected with vector control, wild-type PTPRD, or PTPRD mutants in triplicate using 4 μg of plasmid DNA, 12 μL FuGENE HD (Promega Corporation, Madison, WI), and 200 μL Opti-MEM (Life Technologies, Grand Island, NY) per well added directly to wells containing 3 mL complete medium. After 72 hours, cells were trypsinized and resuspended in PBS containing 0.2% trypan blue (MP Biomedicals, LLC, Santa Ana, CA). Each cell suspension was counted in four fields on a hemacytometer and the average was used to calculate the total number of cells present.

## Results

### PTPRD mutations occur frequently in HNSCC and other cancers

**Fig 1:**
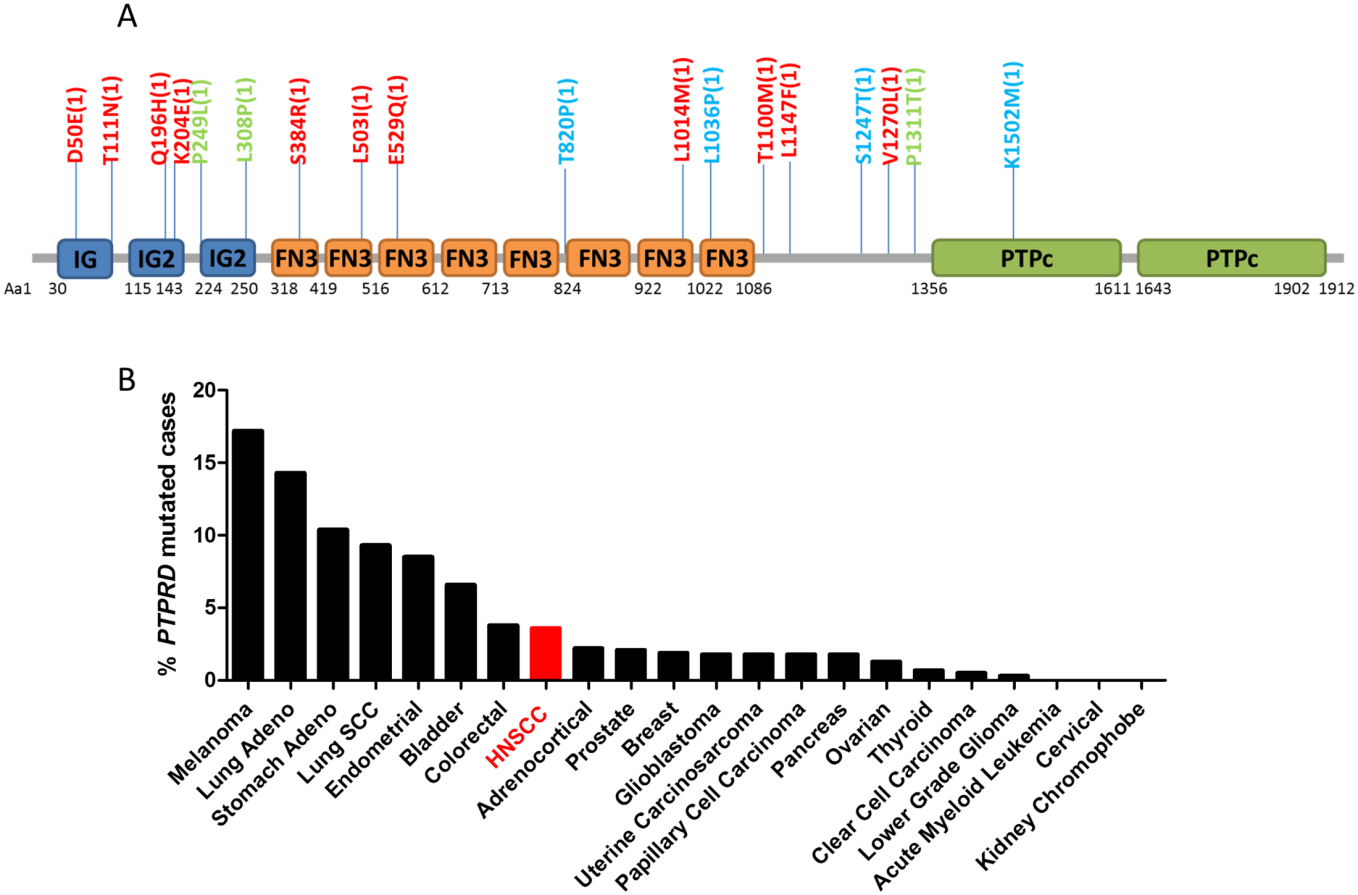
*PTPRD* mutations are common in HNSCC and other cancers. (A) Localization of PTPRD protein mutations reported in HNSCC tumors. Red: TCGA tumors [9,10]; Green: from [6]; Blue: from [7]. IG, Immunoglobulin; IG2, Second immunoglobulin (Ig)-like domain of the receptor protein tyrosine phosphatase (RPTP)-F; FN3, Fibronectin type 3 domain; PTPc, Protein tyrosine phosphatase, catalytic domain. (B) Frequency of *PTPRD* mutations in human cancers as determined by TCGA, with HNSCC shown in red.

Somatic mutation is a common event leading to altered gene and protein function in cancer. Eighteen non-synonymous *PTPRD* mutations have been identified to date in HNSCC tumors. [6,7,9,10] These mutations appear scattered throughout the gene and protein sequence. (Fig 1A) They are all non-recurrent, with each occurring in a single HNSCC tumor, suggesting that these are likely be loss-of-function mutations. Of these 18 mutations, 12 occur in the extracellular domain, 1 is localized to the catalytic domain, and 5 are in the transmembrane region where no conserved domain has been identified. In addition to HNSCC, *PTPRD* mutations are also widely observed in other cancer types. (Fig 1B) In The Cancer Genome Atlas (TCGA) collection, *PTPRD* is most frequently mutated in cutaneous melanoma (59/344 cases, 17.2%), followed by lung adenocarcinoma (33/230 cases, 14.3%), and stomach adenocarcinoma (30/289 cases, 10.4%). [9,10] As in HNSCC, *PTPRD* mutations in these cancers do not appear to cluster into hotspot regions or residues, suggesting that loss of PTPRD function by somatic mutation is a common event across multiple cancer sites.

The only mutation detected in HNSCC that has been previously identified in another cancer is T1100M, which was reported in chronic lymphocytic leukemia. [11] Interestingly, several other mutation sites found in HNSCC have a different amino acid substitution reported in other cancers. For example, while K204E is observed in HNSCC, K204Q has been reported in esophageal cancer. [12] In addition, a review of the COSMIC database (http://cancer.sanger.ac.uk/cancergenome/projects/cosmic/) demonstrates detection of L503V in liver cancer (compared to L503I in HNSCC) and L1036M in colon adenocarcinoma (compared to L1036P in HNSCC). Ding *et al* additionally reported a mutation at this site (L1036Q) in lung adenocarcinoma. [13] Interestingly, while K1502M is the only catalytic domain mutation identified to date in HNSCC, TCGA has identified a K1502* nonsense mutation in lung adenocarcinoma. Together, these findings suggest that while the specific *PTPRD* mutations found to date in HNSCC are unique, the amino acid sites at which they occur may represent important residues that are susceptible to genetic alterations. Indeed, multiple sequence alignment analysis indicates that these residues are highly conserved across species, suggesting they may have a critical role in proper PTPRD function (analysis not shown).

### HNSCC-derived PTPRD mutants lead to increased cell growth

**Fig 2:**
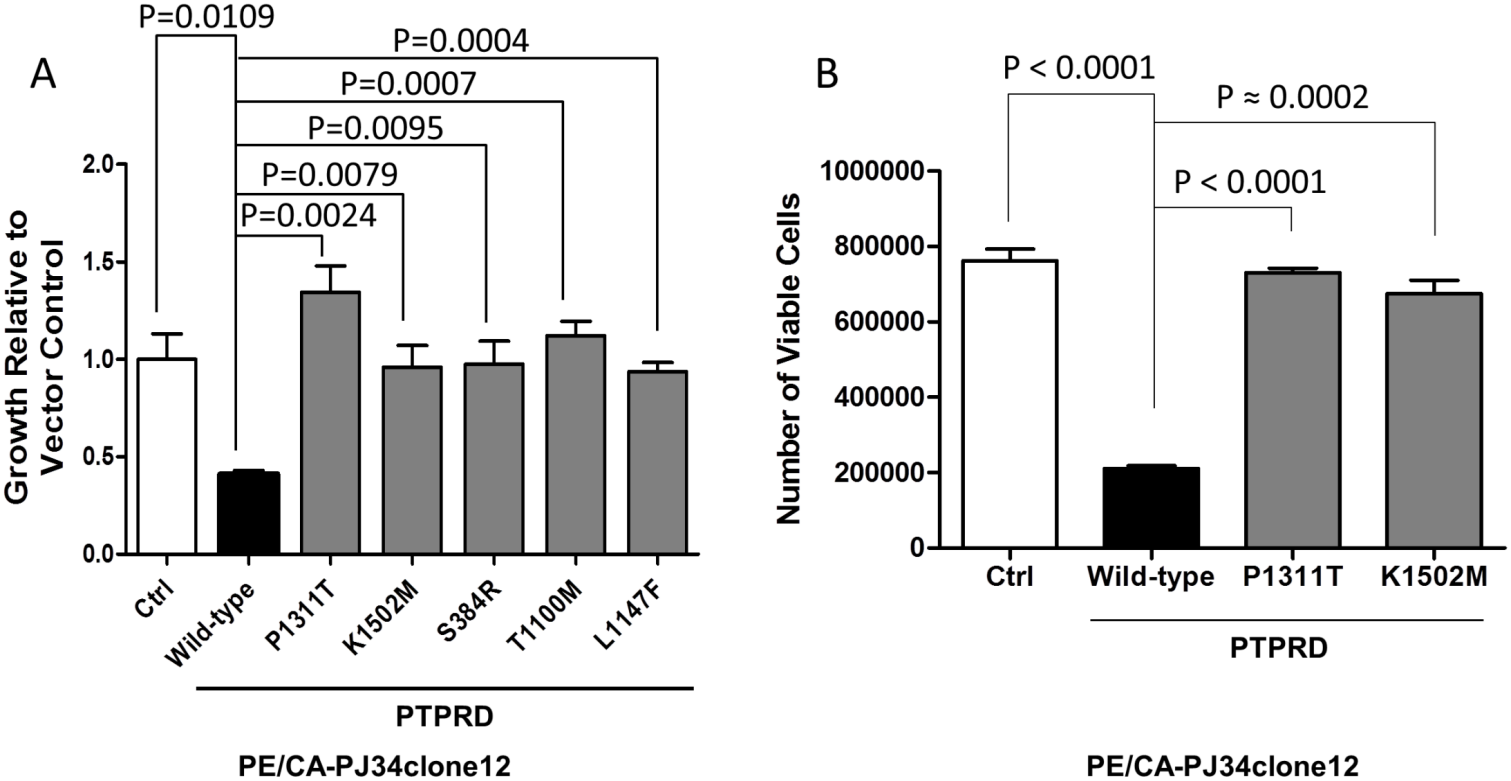
HNSCC-derived *PTPRD* mutations lead to increased cell growth/proliferation in HNSCC cells. (A) Cell growth was assessed by MTT assay 48 hrs after transfection and normalized to vector-transfected controls. One-way ANOVA P = 0.0007. Depicted P values represent the results of pairwise two-tailed unpaired t tests. The experiment was performed three times with similar results. (B) Cell proliferation was assessed by trypan blue exclusion assay 72 hrs after transfection. One-way ANOVA P < 0.0001. Depicted P values represent the results of pairwise two-tailed unpaired t tests.

PTPRD has been reported to serve as a tumor suppressor in human cancer. [3,5,6] To determine the functional consequences of *PTPRD* mutation in HNSCC, we generated several representative HNSCC-derived PTPRD mutants by site-directed mutagenesis, including one mutation in the extracellular domain (S384R), one in the catalytic domain (K1502M), and two in the transmembrane region (T1100M and L1147F). Transient overexpression of these constructs in a HNSCC cell line with no endogenous *PTPR* family mutations (PE/CA-PJ34clone12) revealed that all of the PTPRD mutants tested led to increased growth as determined by MTT assay relative to PTPRD wild-type-transfected cells. (Fig 2A) MTT assay results were further confirmed with representative mutants by trypan blue exclusion assay in PE/CA-PJ34clone12 cells (Fig 2B). Collectively, these results suggest that *PTPRD* mutations inactivate the tumor suppressive function of PTPRD irrespective of their localization throughout the gene, leading to increased growth/proliferation in HNSCC cells.

### PTPRD mutation is associated with increased pSTAT3 expression

**Fig 3:**
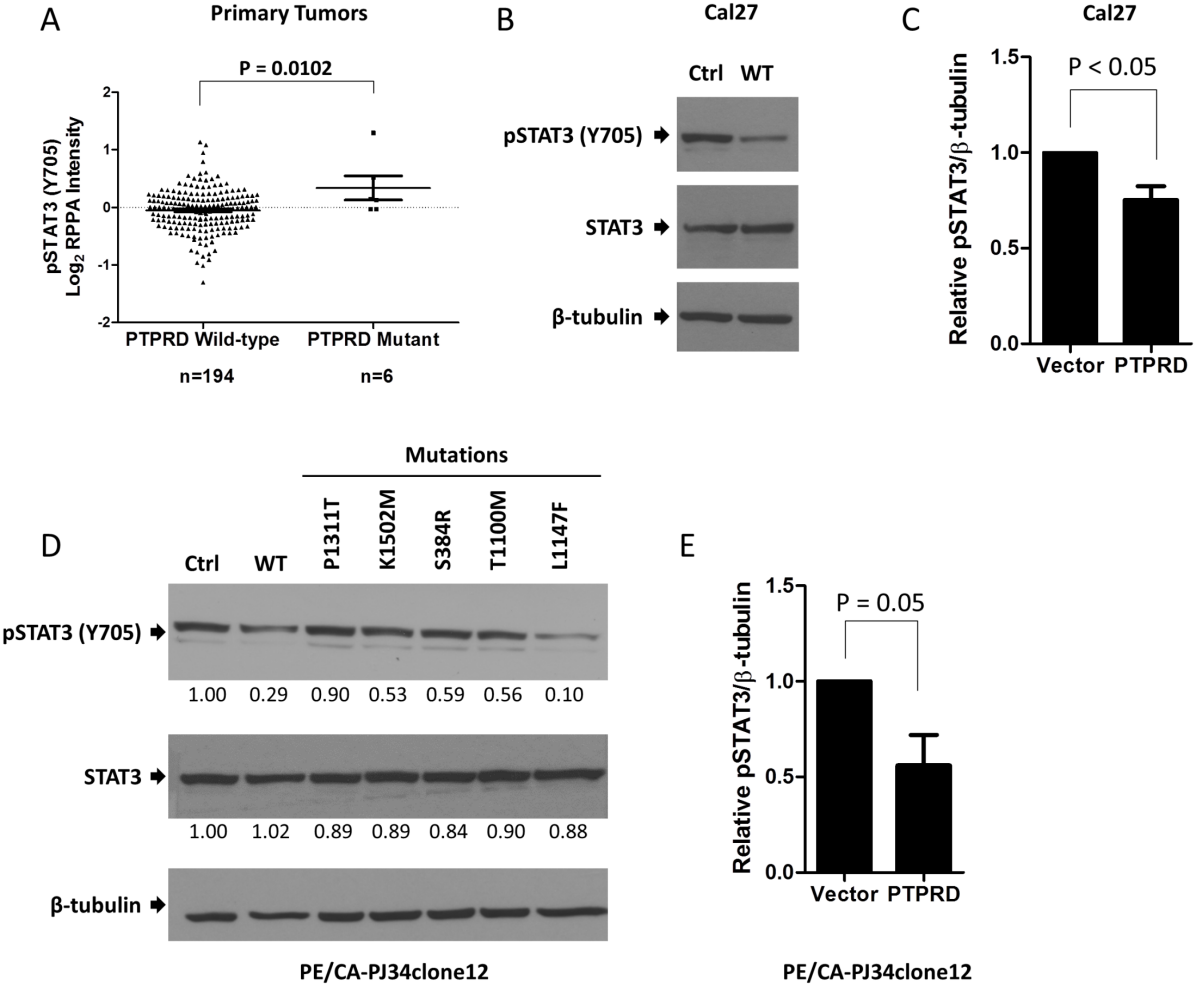
*PTPRD* mutation is associated with increased pSTAT3 (Y705) expression in HNSCC tumors and cell lines. (A) HNSCC tumors harboring *PTPRD* mutations express increased pSTAT3 (Y705) relative to *PTPRD* wild-type tumors as determined by whole exome sequencing and reverse-phase protein array (RPPA). Two-tailed unpaired t test. (B) Overexpression of wild-type PTPRD in a *PTPRD*-mutant cell line (Cal27) leads to decreased pSTAT3 (Y705) expression (C) Quantitation of three replicate experiments as shown in B. Two-tailed unpaired t test. (D) *PTPRD*-wild-type HNSCC cells (PE/CA-PJ34clone12) transiently overexpressing mutant PTPRD exhibit increased pSTAT3 (Y705) expression relative to wild-type-expressing cells. (E) Quantitation of three replicate experiments as shown in D. Two-tailed unpaired t test.

STAT3 is hyper-activated by constitutive phosphorylation of tyrosine 705 (Y705) in many cancer types, including HNSCC. [14,15] Importantly, pSTAT3 (Y705) is a known direct substrate of PTPRD. [6] To determine the effect of *PTPRD* mutation on STAT3 phosphorylation in HNSCC, we first examined 200 HNSCC tumors with both whole exome sequencing and RPPA analyses performed by TCGA and The Cancer Proteome Atlas (TCPA). HNSCC tumors with non-synonymous *PTPRD* mutations express significantly higher levels of pSTAT3 (Y705) relative to tumors with wild-type *PTPRD* (Fig 3A). Notably, *PTPRD* is the only *PTPR* family member for which this correlation is observed. To test the association between *PTPRD* mutation and pSTAT3 expression directly, we transfected an HNSCC cell line with known endogenous *PTPRD* mutation (Cal27 harboring mutation S387L) with wild-type *PTPRD* or vector control and found that overexpression of wild-type PTPRD leads to significantly decreased pSTAT3 expression (P ≤ 0.05). (Fig 3B and 3C) Furthermore, overexpression of mutant PTPRD in HNSCC cells without endogenous *PTPR* family mutations (PE/CA-PJ34clone12) leads to increased pSTAT3 (Y705) expression relative to PTPRD wild-type-overexpressing cells for nearly all mutations tested. (Fig 3D and 3E) Together, these results demonstrate that *PTPRD* mutation leads to increased STAT3 signaling in HNSCC cells and tumors.

### HNSCC cells with endogenous PTPRD mutation are more sensitive to STAT3 pathway inhibition

**Fig 4:**
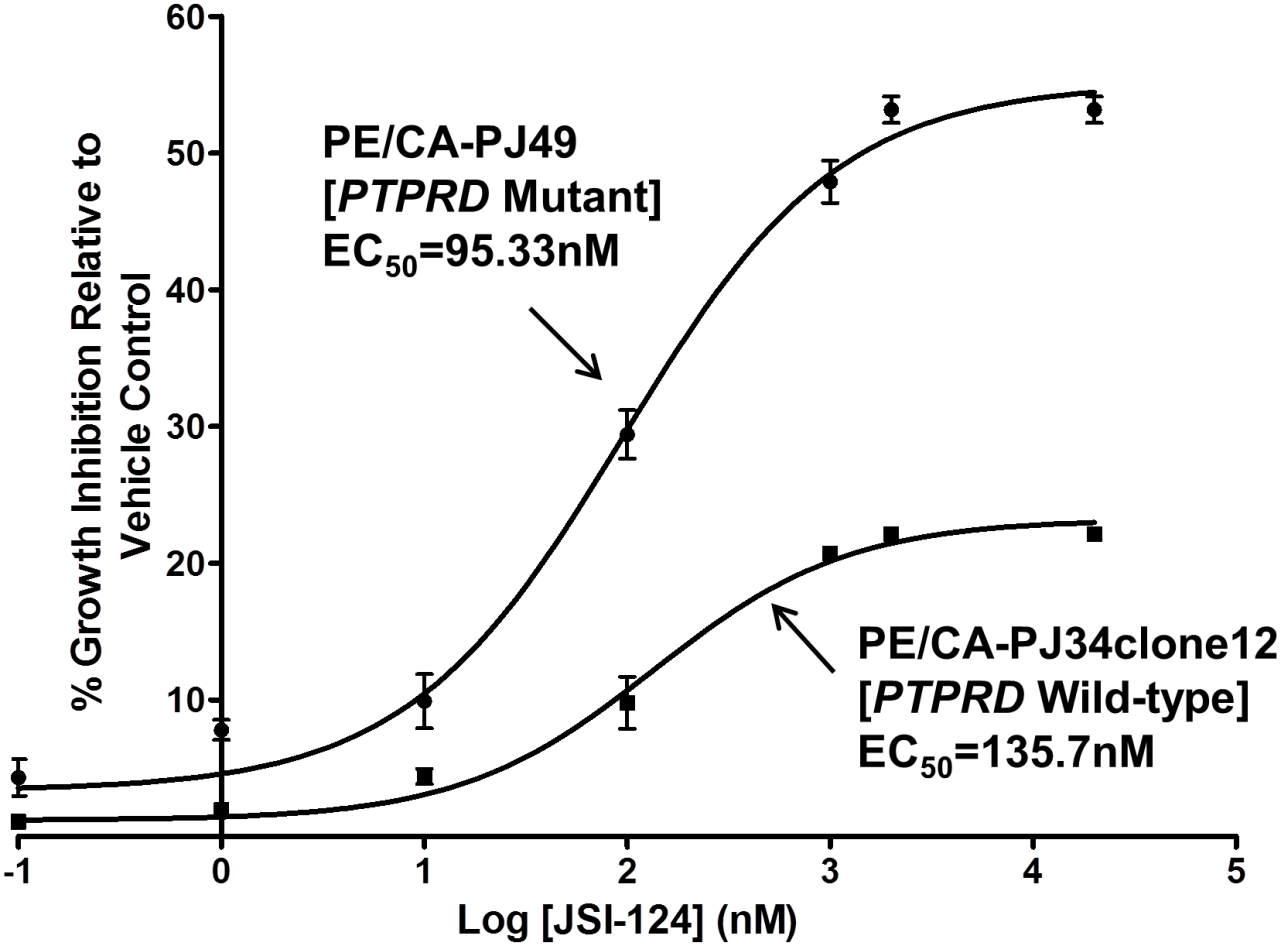
HNSCC cells harboring an endogenous *PTPRD* mutation (PE/CA-PJ49) are more sensitive to the STAT3 pathway inhibitor JSI-124 relative to representative *PTPRD* WT HNSCC cells (PE/CA-PJ34clone12). Cells were treated with increasing concentrations of JSI-124 for 24 hours followed by MTT assay. The experiment was performed three times with similar results.

Since *PTPRD* mutations increase STAT3 activation in HNSCC, we next sought to determine if *PTPRD* mutation leads to increased sensitivity to STAT3 pathway inhibition. To test our hypothesis, we employed HNSCC cells that harbor an endogenous *PTPRD* mutation (PE/CA-PJ49). Treatment with the JAK/STAT inhibitor JSI-124 revels that these *PTPRD* mutant cells exhibit enhanced sensitivity relative to *PTPR* family wild-type cells (PE/CA-PJ34clone12), suggesting that *PTPRD* mutation may serve as a predictive biomarker for response to STAT3 pathway inhibitors (Fig 4).

### PTPRD mRNA expression does not correlate with pSTAT3 expression

Promoter hypermethylation and gene copy number loss represent two additional mechanisms that may lead to loss of function of *PTPRD* via downregulation of mRNA expression. Importantly, *PTPRD* mRNA expression does not correlate with pSTAT3 expression in TCGA HNSCC samples, suggesting that methylation and copy number loss do not significantly contribute to STAT3 overactivation in HNSCC (S1A Fig). Indeed, a more detailed analysis reveals that while *PTPRD* methylation does correlate with mRNA expression as would be expected for any gene (S1B Fig), we observe no correlation between methylation and pSTAT3 expression (S1C Fig), nor do we observe any instances of aberrant promoter hypermethylation (defined here as a methylation level greater than three standard deviations above the mean methylation of the same genetic locus in organ-matched normal samples). In order to validate these findings, we performed methylation-specific PCR on an independent cohort of HNSCC tumors and found evidence of *PTPRD* promoter methylation in 75% of tumors analyzed (30/40) (representative analysis in S2 Fig). Importantly, we also observed *PTPRD* promoter methylation in five paired normal oral mucosa samples from these HNSCC patients (S3 Fig), further suggesting that the *PTPRD* methylation observed in HNSCC is not tumor-specific.

**Fig 5:**
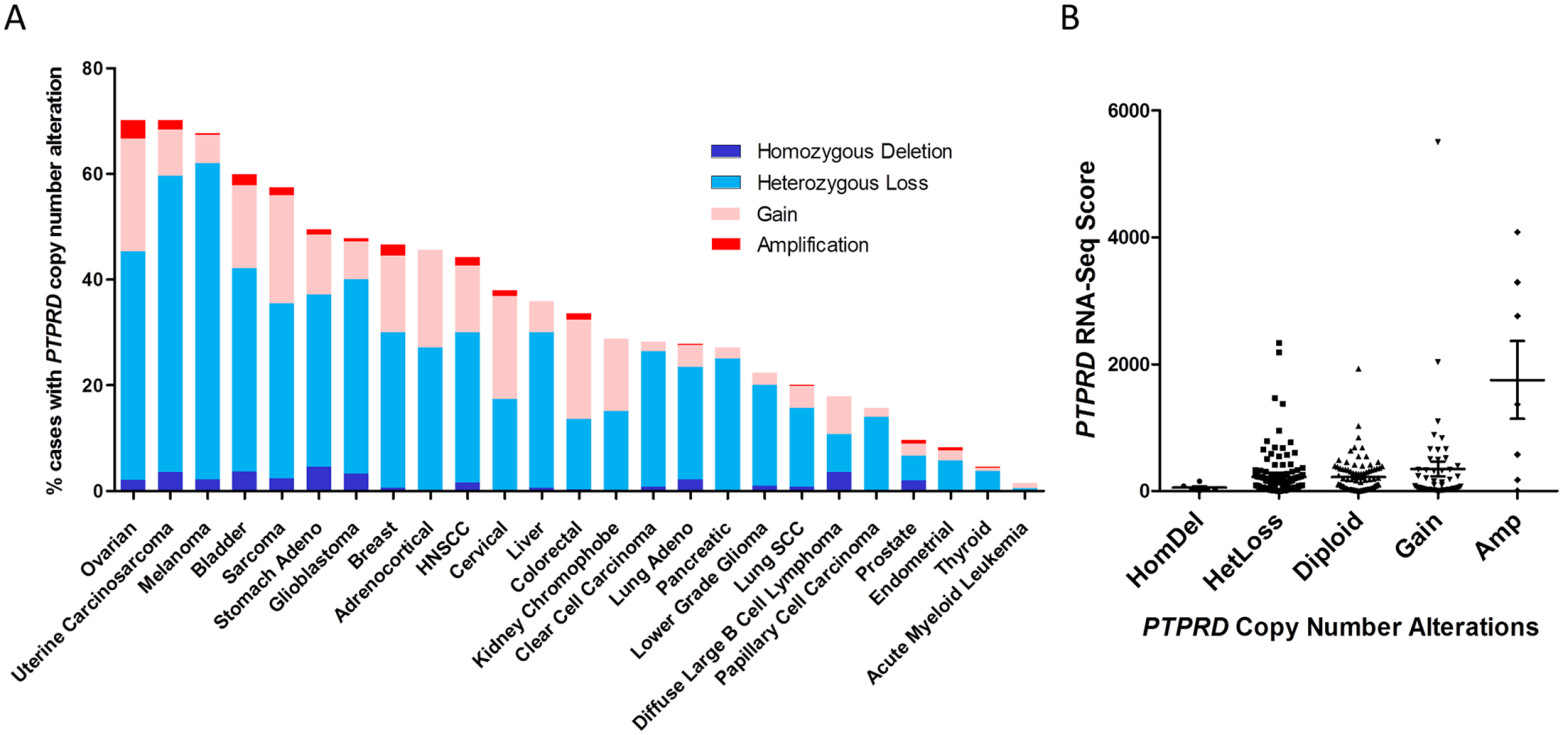
*PTPRD* copy number alterations are frequent across cancers but are not associated with *PTPRD* mRNA expression in HNSCC. (A) Copy number alteration of *PTPRD* in human cancers as determined by TCGA. (B) *PTPRD c*opy number alterations do not correlate with altered *PTPRD* mRNA expression in HNSCC. A Jonckheere-Terpstra test was performed using StatXact software (Cytel, Cambridge, MA).

*PTPRD* copy number alterations occur to a substantial degree in HNSCC and other cancers (Fig 5A), where gene copy loss, particularly heterozygous loss, occurs more frequently than gain in HNSCC and all cancers analyzed with the exception of colorectal and cervical cancers. Importantly, we do not observe a significant correlation between copy number alterations and mRNA expression in HNSCC (Fig 5B). Together, these findings suggest that promoter hypermethylation and gene copy number loss do not significantly contribute to STAT3 overactivation in HNSCC.

## Discussion

STAT3 is an oncogene that is hyper-activated in many cancers, including HNSCC, where STAT3 hyper-activation is associated with decreased survival and emergent resistance to EGFR-targeted therapy. [9,16,17] Potential mechanisms that may result in increased STAT3 activation in cancer include increased signaling through upstream receptor or non-receptor tyrosine kinases such as EGFR and JAK, and/or inactivation of negative regulators of STAT3, including protein tyrosine phosphatases. [18,19] We recently reported that the receptor-type protein tyrosine phosphatase (PTPR) family is frequently mutated in HNSCC and other cancers and demonstrated that HNSCC-derived mutations of PTPRT induce STAT3 phosphorylation and drive HNSCC cell survival. [4] As PTPRD represents an additional receptor-like phosphatase that directly targets STAT3, we sought to determine if genetic or epigenetic loss of PTPRD function may contribute to STAT3 overactivation in HNSCC.

In the present study, we first identified 18 previously uncharacterized *PTPRD* mutations in HNSCC tumors. These mutations are all non-recurrent and are located throughout gene, a pattern that is consistent with that generally observed for tumor suppressor genes. We have shown that overexpression of wild-type PTPRD leads to growth suppression in HNSCC cells, while overexpression of representative mutants does not, thus establishing a functional consequence of *PTPRD* mutation in HNSCC models. These findings are consistent with those of previous *in vitro* studies across several cancer types, including glioblastoma, melanoma, colorectal cancer, and neuroblastoma, where wild-type PTPRD has been observed to suppress colony formation and cell growth as well as enhance apoptosis, while cancer-derived PTPRD mutants do not. [3,6,20] These cumulative findings suggest that *PTPRD* mutations in cancer lead to loss of its tumor suppressive function.

In order to determine if *PTPRD* mutation affects the activity of STAT3, a PTPRD substrate, we analyzed TCGA and TCPA data and found that HNSCC tumors with *PTPRD* mutations express significantly elevated pSTAT3 (Y705) relative to *PTPRD*-wild-type tumors. We previously reported a similar association between pSTAT3 (Y705) expression and mutation of a group of putative *PTPR* tumor suppressor genes, including *PTPRD*. [4] When each gene is considered individually rather than as a group, *PTPRD* is the only *PTPR* family member for which mutation is significantly associated with pSTAT3 (Y705) expression (S4 Fig). This result suggests that while the number of tumors harboring a mutation in any single *PTPR* family member may be insufficient to detect a significant difference in pSTAT3 (Y705) expression, *PTPRD* mutation in particular is uniquely associated with an increase in pSTAT3 (Y705) expression that is sufficiently large to detect statistical significance. Futhermore, overexpression of wild-type PTPRD in HNSCC cell lines harboring endogenous *PTPRD* mutations leads to downregulation of pSTAT3 (Y705), thus confirming that PTPRD regulates STAT3 activation in HNSCC models. While wild-type PTPRD leads to downregulation of pSTAT3 (Y705) in HNSCC cells, overexpression of most HNSCC-derived mutants does not alter pSTAT3 (Y705) expression relative to vector control, suggesting that these mutations lead to loss of function, but not in a dominant negative manner. In the case of the L1147F mutation tested herein, overexpression in HNSCC cells leads to increased growth, but not increased pSTAT3 (Y705) expression (see Figs 2A and 3D), indicating that certain mutations may lead to cancerous phenotypes in a STAT3-independent manner. These mutations may manifest through alternate mechanisms which may include extracellular interactions or alteration of relative affinities for alternate enzymatic substrates.

As *PTPRD* mutation leads to increased STAT3 activation in HNSCC, we next tested whether cells harboring a *PTPRD* mutation may be more sensitive to STAT3 pathway inhibition. Here we demonstrate that HNSCC cells with an endogenous *PTPRD* mutation are more sensitive to the JAK/STAT inhibitor JSI-124 than HNSCC cells harboring no *PTPR* family mutations, suggesting that HNSCC tumors with *PTPRD* mutations may be exquisitely sensitive to STAT3 inhibitors that are currently in preclinical and clinical development. In order to devise an optimal treatment strategy for HNSCC patients with *PTPRD* mutations, further study of additional PTPRD-interacting proteins that may serve as therapeutic targets may be warranted. In particular, *PTPRD* mutation may additionally confer sensitivity to aurora kinase A inhibitors currently in clinical development, where aurora kinase A phosphorylation and stability are normally regulated by PTPRD. [3]

An additional mechanism by which *PTPRD* function may be lost is through mRNA downregulation, including by promoter hypermethylation or gene copy number loss. First, we determined that *PTPRD* mRNA expression does not correlate with pSTAT3 expression in HNSCC, suggesting that these mechanisms are not likely to significantly contribute to STAT3 overactivation in HNSCC. It should be noted that this analysis may be complicated by the several cases in which little or no *PTPRD* mRNA was detected. Indeed, our overexpression studies in HNSCC cells suggest that expression of PTPRD would be expected to impact pSTAT3 (Y705) expression. Nonetheless, this correlation has not emerged in the HNSCC tumors analyzed to date.

A more detailed analysis of these mechanisms next revealed that the *PTPRD* promoter is not hypermethylated in HNSCC relative to organ-matched normal tissue, a finding we validated in an independent cohort of HNSCC tumor and matched normal pairs by methylation-specific PCR. The disparity between our present findings and previous reports of *PTPRD* promoter methylation in HNSCC is likely due to the prior lack of comparison between tumor and normal tissue. [6] The low level of *PTPRD* promoter methylation observed in HNSCC is additionally not associated with altered pSTAT3 (Y705) expression, further suggesting that this event does not significantly contribute to the cancer phenotype in HNSCC. A similar lack of aberrant *PTPRD* promoter hypermethylation has also been reported in cutaneous squamous cell carcinoma, suggesting this may be an uncommon event in multiple epithelial malignancies. [21]

Our analysis of TCGA data indicates that nearly half of HNSCC tumors harbor a copy number alteration of *PTPRD* and that copy number loss is more frequent than copy number gain in HNSCC and across nearly all cancers analyzed. *PTPRD* homozygous or heterozygous deletion has also been reported in cutaneous squamous cell carcinoma [21,22], GBM [6,20,23,24], lung cancer [25,26], neuroblastoma [27], metastatic melanoma [28,29], squamous cell carcinoma of the vulva [30], hepatocellular carcinoma [31], and laryngeal squamous cell carcinoma [5]. Importantly, no significant association was detected between *PTPRD* copy number alteration and *PTPRD* mRNA expression in HNSCC, suggesting that copy number loss is not the primary mechanism of loss of PTPRD function in HNSCC.

In conclusion, we have demonstrated herein that somatic mutation of *PTPRD* is the primary mechanism by which PTPRD loss of function occurs in HNSCC. We propose that nearly all of these mutations lead to loss of catalytic activity and hence reduced dephosphorylation of the substrate pSTAT3 (Y705). While promoter methylation and copy number loss are detectable at the *PTPRD* locus, these events are not associated with upregulation of pSTAT3 (Y705) expression. In contrast, somatic mutation of *PTPRD* leads to increased STAT3 activation in HNSCC tumors and cell lines, concomitant with increased cell growth and sensitivity to STAT3 pathway inhibition. These findings suggest that *PTPRD* mutation may represent a predictive biomarker for exquisite response to STAT3 targeted therapy in HNSCC.

## Supporting Information

S1 Fig *PTPRD* promoter methylation correlates with *PTPRD* mRNA expression but does not correlate with pSTAT3 (Y705) expression in HNSCC. (A) *PTPRD* mRNA expression is not significantly associated with pSTAT3 (Y705) expression. n = 172, Pearson r = 0.05157, P = 0.5017, R^2^ = 0.002659. (B) *PTPRD* promoter methylation correlates with *PTPRD* mRNA expression. n = 172, Pearson r = -0.5242, P < 0.0001, R^2^ = 0.2747. (C) *PTPRD* promoter methylation is not significantly associated with pSTAT3 (Y705) expression. n = 211, Pearson r = 0.0008795, P = 0.9899, R^2^ = 7.735e-007.
(TIF)

S2 Fig MSP analysis of representative HNSCC tumor samples. Methylation was observed in 30/40 (75%) HNSCC tumors analyzed. M denotes primers amplifying methylated sequences, while U denotes primers amplifying unmethylated sequences.
(TIF)

S3 Fig The *PTPRD* promoter is not hyper-methylated in HNSCC tumors compared with adjacent normal mucosa. Five HNSCC tumors and matched normal mucosa from the same patients were collected and analyzed by MSP. M denotes primers amplifying methylated sequences, while U denotes primers amplifying unmethylated sequences.
(TIF)

S4 Fig *PTPRD* is the only family member for which mutation is associated with pSTAT3 (Y705) expression in HNSCC. Whole exome sequencing and reverse-phase protein array data reveal that no other *PTPR* family mutations are significantly associated with pSTAT3 (Y705). P values represent the results of two-tailed unpaired t tests. N/A indicates insufficient sample size for calculation.
(TIF)
