# Supplementary figures and images for: Loss-of-Function PTPRD Mutations Lead to Increased STAT3 Activation and Sensitivity to STAT3 Inhibition in Head and Neck Cancer
Source: PLoS One. 2015 Aug 12;10(8):e0135750. doi: 10.1371/journal.pone.0135750 (PMC4534317; doi:10.1371/journal.pone.0135750)

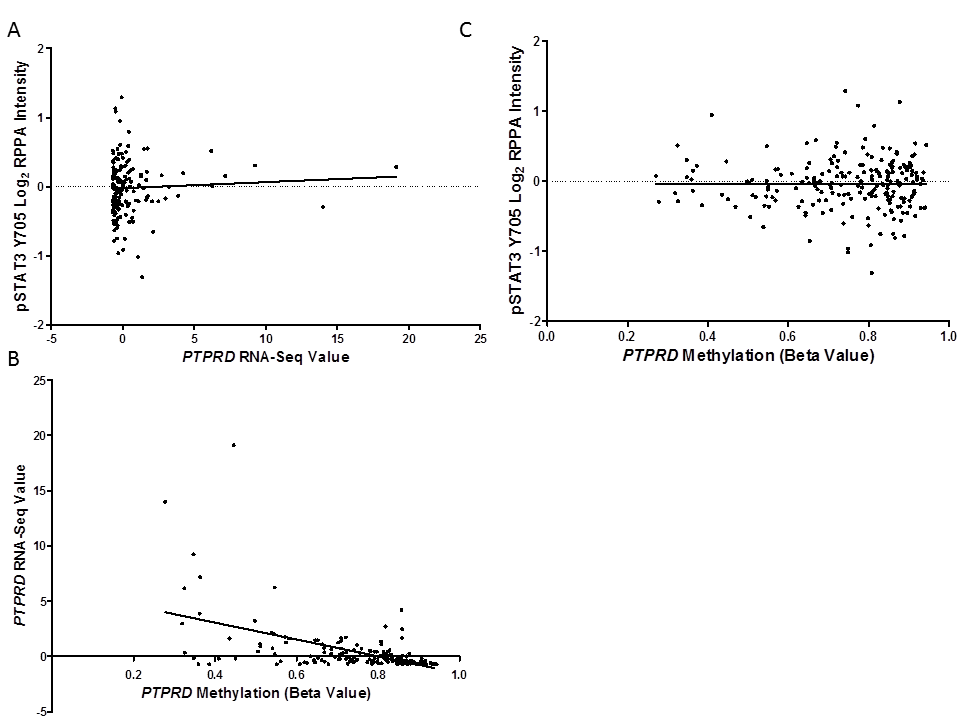

Supplement: S1 Fig — (A) PTPRD mRNA expression is not significantly associated with pSTAT3 (Y705) expression. n = 172, Pearson r = 0.05157, P = 0.5017, R2 = 0.002659. (B) PTPRD promoter methylation correlates with PTPRD mRNA expression. n = 172, Pearson r = -0.5242, P < 0.0001, R2 = 0.2747. (C) PTPRD promoter methylation is not significantly associated with pSTAT3 (Y705) expression. n = 211, Pearson r = 0.0008795, P = 0.9899, R2 = 7.735e-007. (TIF) [file pone.0135750.s001.tif]

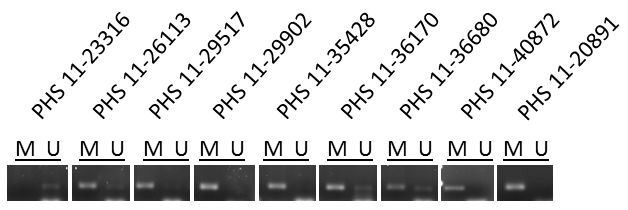

Supplement: S2 Fig — Methylation was observed in 30/40 (75%) HNSCC tumors analyzed. M denotes primers amplifying methylated sequences, while U denotes primers amplifying unmethylated sequences. (TIF) [file pone.0135750.s002.tif]

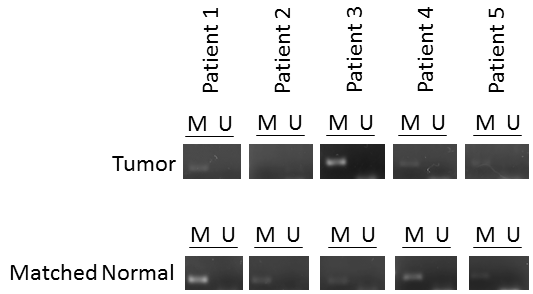

Supplement: S3 Fig — Five HNSCC tumors and matched normal mucosa from the same patients were collected and analyzed by MSP. M denotes primers amplifying methylated sequences, while U denotes primers amplifying unmethylated sequences. (TIF) [file pone.0135750.s003.tif]

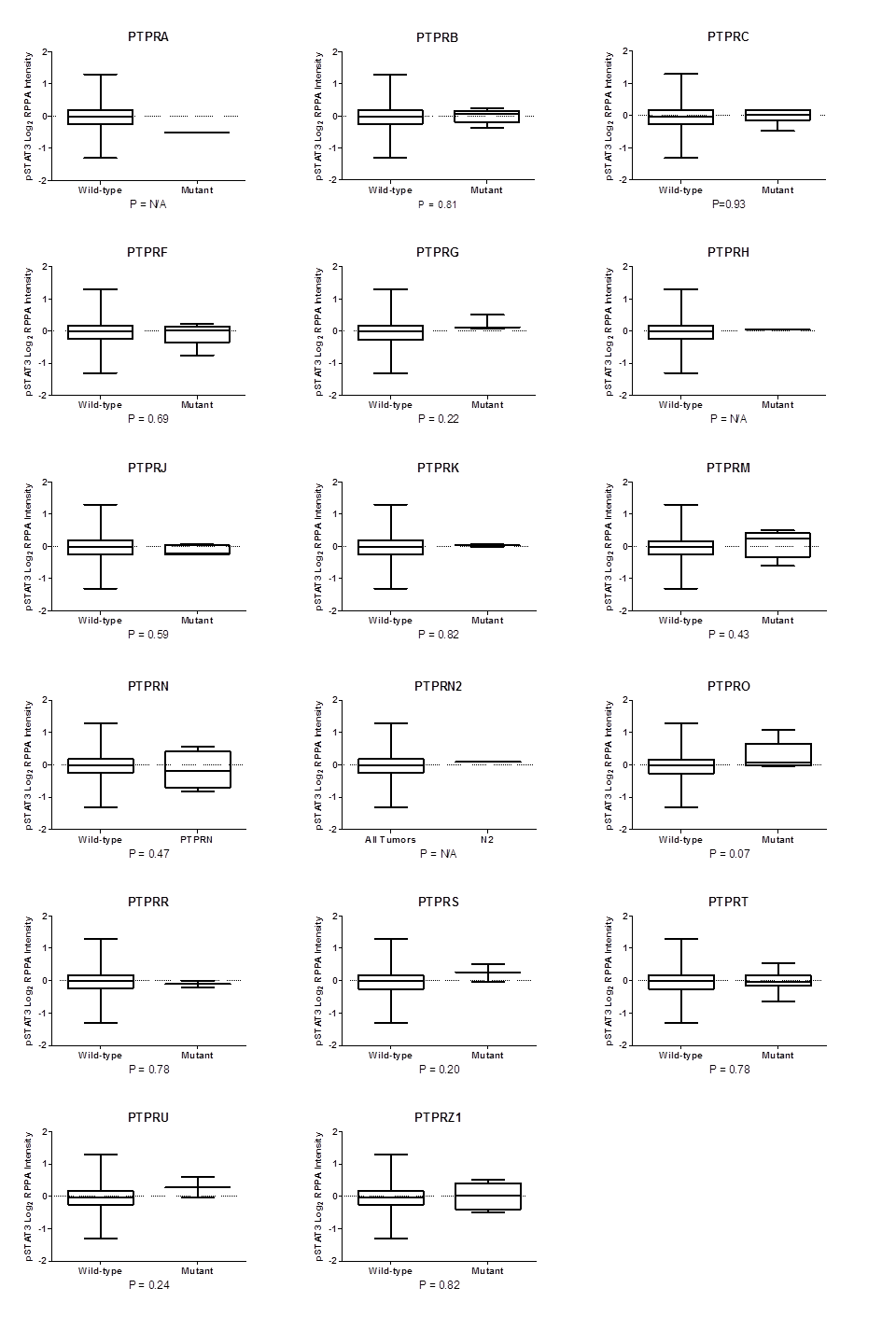

Supplement: S4 Fig — Whole exome sequencing and reverse-phase protein array data reveal that no other PTPR family mutations are significantly associated with pSTAT3 (Y705). P values represent the results of two-tailed unpaired t tests. N/A indicates insufficient sample size for calculation. (TIF) [file pone.0135750.s004.tif]
